# Infection of Multiple Tunneled Dialysis Catheters Resulting From the Contamination of the Chlorhexidine Solution by Serratia marcescens

**DOI:** 10.7759/cureus.45693

**Published:** 2023-09-21

**Authors:** Joana Gomes Cochicho, José Miguel Silva, Marcio Viegas

**Affiliations:** 1 Internal Medicine Department, Hospital Doutor José Maria Grande, Portalegre, PRT; 2 Hemodialysis Department, Centro de Portalegre, Fundação Renal Portuguesa, Portalegre, PRT; 3 Nephrology Department, Hospital Doutor José Maria Grande, Portalegre, PRT

**Keywords:** hemodialysis access, chlorohexidine colonization, tunnelled dialysis catheter infection, hemodialysis, serratia marcescens

## Abstract

Tunnelled dialysis catheters continue to be a choice in several patients as hemodialysis access. According to Kidney Disease Outcomes Quality Initiative guidelines, its handling implies disinfection, that can be performed using chlorhexidine solutions. Theoretically, these solutions have bactericidal capacity at concentrations greater than 0.12%.

We present a curious situation of failure of the antiseptic process due to contamination of the chlorhexidine solution 4% of aqueous base. In this hemodialysis clinic, three cases of infections by the bacteria *Serratia marcescens* were identified over 2 weeks - in two of the cases, identified in blood culture, and in the other case in the exudate from the exit site of the catheter.

Considering the abnormal number of infections by this agent and the fact that these patients were on different shifts, were treated in different rooms, and handled by different nurses, the antiseptic solutions used in the different hemodialysis rooms were analyzed, as well as a closed package from the same batch. After microbiological tests were performed on the antiseptic solution, we identified the growth of *Serratia marcescens*. This result identified the culprit as being the contamination of the 4% chlorhexidine solution.

The competent authorities were notified, and the disinfection method was changed to use a chlorhexidine alcohol-based solution.

## Introduction

Tunnelled dialysis catheters (TDCs) are not the primary choice for dialysis access due to the associated morbidity. Nevertheless, they remain a viable option for numerous patients, primarily utilized in cases of non-maturing arteriovenous access, delayed arteriovenous vascular access construction, or the absence of alternative access options [1].

Infections, particularly bacteremia, represent a serious complication associated with the use of TDCs [2]. 

To prevent such occurrences, it is recommended to perform disinfection of the TDC exit site using a chlorhexidine solution or an alcohol solution during manipulation, including bandage replacement [3].

Chlorhexidine, being a cationic molecule, binds indiscriminately to the phospholipid membrane of bacteria, resulting in an alteration of the osmotic balance within the cell wall. This disturbance causes the release of low molecular weight components. While this process can be reversible at lower concentrations, at higher concentrations, it leads to cytolysis. As a result, the effect of chlorhexidine is dose-dependent, with lower concentrations exhibiting bacteriostatic activity and higher concentrations displaying bactericidal activity, typically exceeding 0.12% [4].

This article aims to report a series of cases of *Serratia marcescens* (*S. marcescens*) infection due to the contamination of the chlorhexidine solution.

## Case presentation

Hemodialysis clinic description

The hemodialysis clinic is a facility that consists of four hemodialysis rooms. The clinic accommodates four shifts, with approximately 20 patients in each shift. These patients undergo three hemodialysis sessions of four hours per week.

Adaptation of the catheter dressing handling procedure during the COVID-19 pandemic period

Before the COVID-19 pandemic period, in several patients, impregnation of the catheter dressing with an alcoholic solution (Cutasept®, Paul Hartmann AG, Heidenheim, Germany) was used to help detach the dressing. Due to the shortage of alcohol experienced at the beginning of the pandemic period, this step was changed. The alcoholic solution previously used in this process was replaced by a saline solution. The disinfection of the exit site with a 4% chlorhexidine solution was maintained.

During a two-week period in April 2020, an unusual number of *S. marcescens* infections were detected in three patients. These patients were attended to in different rooms, on different shifts, by different nurses. The details of these cases are outlined below and summarized in Table 1.

**Table 1:** Summary of infectious characteristics and instituted therapy * Microbiological analysis was performed using blood agar. TDC: Tunnelled dialysis catheter

	Symptoms	Microbiological analysis*	Antibiotic susceptibility test	Diagnosis	Treatment
Case 1	Fever and shivering	S. marcescens detected in hemoculture	Sensitive to piperacillin + tazobactam, cefepime, ceftazidime, ciprofloxacin. Resistant to amoxicillin + clavulanic acid.	Bacteremia originating from catheter-associated infection	Vancomycin and Gentamicin; Ciprofloxacin; Exeresis of catheter.
Case 2	Fever and shivering	S. marcescens detected in hemoculture	Sensitive to ciprofloxacin and cefotaxime. Resistant to gentamicin, amoxicillin + clavulanic acid and cefuroxime.	Bacteremia originating from catheter-associated infection	Vancomycin and Gentamicin; Ciprofloxacin; Ceftazidime; Exeresis of catheter.
Case 3	Fever and shivering	S. marcescens detected in exudate from the site of TDC insertion	Sensitive to piperacilin + tazobactam, cefotaxim, ceftazidime, gentamicin, ciprofloxacin. Resistant to amoxicillin + clavulanic acid.	Catheter-associated infection	Vancomycin and Gentamicin; Ciprofloxacin; Exeresis of catheter.

Case 1

An 83-year-old male patient with stage 5 chronic kidney injury secondary to diabetic nephropathy, with medical records of type 2 diabetes mellitus, arterial hypertension, prostatic disease, bilateral cataracts, and Meniere's syndrome.

The patient started hemodialysis in 2019.

In April 2020, the active vascular access was an arteriovenous fistula with a recent puncture onset. For that reason, the patient maintained the TDC in the right internal jugular vein, which was submitted to a regular disinfection procedure.

By that time, the patient developed a fever (tympanic temperature of 38ºC) accompanied by shivering. Hemodynamically stable, the patient did not exhibit any signs of inflammation at the TDC exit site, nor any other indications of an infectious source. The patient commenced empiric antibiotic therapy with vancomycin and gentamicin, and blood cultures were performed. It allowed for the isolation of *S. marcescens*. The antibiotic was adjusted to ciprofloxacin according to the antibiogram and the TDC was removed.

Case 2

A 40-year-old female with stage 5 chronic kidney injury secondary to lupus nephritis, with a history of systemic lupus erythematosus, antiphospholipid antibody syndrome, complications of prolonged corticosteroid therapy, and ischemic heart disease.

Since the age of 16, she has required renal replacement therapy, including peritoneal dialysis for several years, which was suspended due to the development of an abdominal hernia. She subsequently underwent a deceased donor kidney transplant, which was lost due to chronic dysfunction, leading to the recent need for hemodialysis. Due to poor vascular access, after angioplasty of stenosis of the superior cava vein, it was possible to insert a TDC on the right internal jugular vein.

In April 2020, during hemodialysis, the patient presented with fever and chills (tympanic temperature of 38.7ºC). There were no inflammatory signs at the catheter exit site, nor other symptoms suggestive of an infectious focus.

The patient was empirically treated with vancomycin and gentamicin. Blood cultures were performed, and *S. marcescens* was isolated. After antibiotic susceptibility testing, the antibiotics were replaced with ciprofloxacin. Initially, the TDC was not removed due to poor vascular heritage.

However, the patient experienced a recurrence of bacteremia, twice, during the following 4 months, requiring a second cycle of ciprofloxacin and a third one with ceftazidime. Antibiotic therapy was based on the antibiotic susceptibility test results in both cases. The patient underwent an echocardiogram that did not reveal any evidence of endocarditis. However, the definitive resolution of the inferior implied the excision and replacement of the TDC.

Case 3

A 35-year-old male with stage 5 chronic kidney injury secondary to ischemic acute tubular necrosis, with a personal history of hypothyroidism, depressive syndrome, post-nephrectomy of the left kidney due to sarcoma of this organ, and multiple post-surgical complications.

He had been undergoing hemodialysis since the postoperative period in April 2019.

The active vascular access was an arteriovenous fistula with a recent puncture onset. For that reason, the patient maintained the TDC in the right internal jugular vein, which was submitted to a regular disinfection procedure.

In April 2020, during a hemodialysis session, the patient presented with a fever (tympanic temperature of 38.1ºC) and chills. A mild purulent exudate was identified at the site of TDC exit. Microbiological analysis of this exudate identified *S. marcescens*. Blood cultures were not obtained due to logistical constraints. The patient started treatment with vancomycin and gentamicin, and an excision of the TDC was performed. The antibiotic was adjusted to ciprofloxacin according to the antibiogram.

In addition to the documented cases of bacteremia outlined above, there was a noticeable rise in the number of reported symptoms suggestive of potential infections among other patients during their hemodialysis sessions, for which, unfortunately, it was not possible to isolate the agent in cultures, in some cases with evidence of infection at the exit site of the TDC, but without any exudate suitable for collection.

The three documented cases of TDC infection caused by an uncommon bacterium for this source of infection, without an apparent epidemiological link, sparked curiosity and prompted a literature search that led us to investigate possible contamination of the antiseptic solution used.

Microbiological analysis was conducted on samples of the chlorhexidine 4% being utilized in all four hemodialysis rooms. Furthermore, we examined sealed packages from the current batch in use, as well as another batch that had been previously used. The results indicated the presence of *S. marcescens *contamination in all samples.

The relevant authorities were promptly notified about this situation to prevent any further harm or complications. In this clinic, the procedure for disinfecting the TDC was changed to replace the aqueous-based chlorhexidine solution with an alcohol-based chlorhexidine solution.

## Discussion

*S. marcescens* is a Gram-negative bacterium that presents an important capacity to produce biofilms, which contributes to its pathogeny, by decreasing antibiotic penetration and by promoting slow growth rates [5].

This bacterium has the potential to induce infections in various human body systems, including the eyes, lungs, urinary tract, and bloodstream [5]. Additionally, it has been observed in infections associated with central venous catheters [6].

In recent decades, several outbreaks of *S. marcescens* have been identified, some of which were caused by contamination of medical devices and pharmaceutical products [6].

Hervé et al. documented an outbreak caused by *S. marcescens* that occurred in 2014 in a surgical department in Chile. A total of 13 clinical infections were identified, with five of them associated with central venous catheters. Four cases of central venous catheter colonization were documented and six cases of bacteremia without clinical repercussions were also identified. Microbiological analysis of the antiseptic solution, specifically a 2% aqueous chlorhexidine tincture, confirmed the presence of S*. marcescens*. Further investigation revealed contamination of a batch of the antiseptic solution during the production process [7].

Merino et al. reported 21 cases of *S. marcescens* bacteremia detected in multiple hemodialysis clinics within the Comunidad Autónoma de Madrid in 2014 and 2015. They identified several batches of aqua-based chlorhexidine at 0.05% and 2% that were colonized by the same microbiological agent [8].

Subsequently, González et al. [9] documented an outbreak of 14 cases of *S. marcescens* bacteremia associated with catheters in a hemodialysis clinic in Spain. These cases were confirmed through blood cultures and occurred during the same period as the ones reported by Merino et al. [8].

There have been documented instances of bacterial contamination affecting the antiseptic chlorhexidine, with *S. marcescens* being among the reported bacteria involved [10,11].

During a study conducted in 1981 in Canada, in response to an outbreak of *S. marcescens* infections, various packages of chlorhexidine were examined and a significant number of them were found to be contaminated. The bacteria were detected in the liquid, sediment, and on the sides of the bottles. This investigation also highlighted the prolonged survival of *S. marcescens* in chlorhexidine [11].

Later, a plausible explanation was proposed to shed light on the resistance exhibited by a specific strain of *S. marcescens* to chlorhexidine. The suggestion was that a modification in the internal membrane impeded the leakage of potassium following exposure to the antiseptic, providing a probable rationale [12].

After observing the infection of several patients with *S. marcescens* under the previously described circumstances, we began to suspect the possible colonization of the antiseptic being used in the clinic. As a result, a thorough microbiological investigation was conducted on the chlorhexidine solution utilized in each room, as well as an examination of sealed packages. The presence of *S. marcescens* was detected in all analysed samples, thereby confirming the hypothesis of chlorhexidine solution contamination.

Interestingly, the results of the antibiotic sensitivity tests showed a slight variation among different individuals, as shown in Table 1. This variation likely arises from the adaptive mechanisms of antibiotic resistance inherent to this bacterium [13].

It is noteworthy that the scarcity of alcohol during the COVID-19 pandemic prompted a modification in the handling protocol of TDC. Previously, the process included both chlorhexidine and alcohol as previously described. However, during the timeframe relevant to these infections, disinfection was performed solely with 4% chlorhexidine, which, as previously stated, possesses bactericidal properties. However, as has been reported by other research groups, the solution remains vulnerable to contamination by *S. marcescens*.

The authorities were promptly notified about the outbreak of *S. marcescens* infections to prevent any further spread caused by the contaminated product.

In Portugal, products like this antiseptic solution are classified according to the European Regulation (EU) No 528/2012 as biocidal products. Considering the need for using these antiseptic solutions for pre-disinfection before performing invasive procedures according to several guidelines, with a high risk of associated infection, as in the presented cases, we believe that the regulation of these substances should be more rigorous. Therefore, we suggest that the classification of these products be reviewed so that the safety measures in their manufacturing and packaging processes are similar to those required for medicines, making their use safer.

Furthermore, this incident prompted a critical evaluation of the procedural changes implemented during the pandemic, resulting in the introduction of alcohol-based chlorhexidine to enhance the safety of the process in this hemodialysis clinic.

## Conclusions

Considering the described situation and the reported outbreaks involving the contamination of ostensibly sterile products by this bacterium, the clinic decided to switch the TDCs' disinfection method to alcohol-based chlorhexidine.

The purpose of this report is to emphasize the importance of maintaining rigorous quality control measures for non-pharmaceutical products used in clinical procedures.

Furthermore, given the limited number of published cases, we believe that documenting this outbreak can contribute to advancing knowledge concerning this microbiological agent, and subsequently aid in implementing preventive measures to combat its colonization or contamination.
